# Evaluating and upgrading the performance of a bridge network structure with Rayleigh distribution lifetimes

**DOI:** 10.1371/journal.pone.0315845

**Published:** 2025-01-17

**Authors:** Hossam A. Nabwey, Adel A. El-Faheem, Mohammed Ashraf Denguir, A. M. Rashad

**Affiliations:** 1 Department of Mathematics, College of Science and Humanities in Al-Kharj, Prince Sattam bin Abdulaziz University, Al-Kharj, Saudi Arabia; 2 Department of Mathematics, Aswan University, Faculty of Science, Aswan, Egypt; Tanta University, EGYPT

## Abstract

In this work, bridge network model with Rayleigh distribution lifetimes is used. Two main techniques are calculated to upgrade this model: reduction and redundancy techniques. In order to compare the effectiveness of the various approaches, the survival function, the mean time to failure and gamma-fractiles for the original and upgraded model are calculated. Finally, we analyze comprehensively a computer simulation example to distingue between the methods. The numerical simulations were done in Mathematica which indicated that the upgraded system performs better than the original one and the best technique is by cold duplication.

## 1 Introduction

Survival analysis or reliability is a crucial prerequisite to ensure that the product or system will perform effectively during its designated lifetime. Lord Rayleigh proposed in 1880 Rayleigh distribution [1], which its failure rate is increasing function of time. It has important uses in a wide range of scientific domains, including industry, survival data analysis, and medicine. When the failure time began at a specific moment, α = 0, and α stands for the item’s guarantee; that is, the parameter α describes the time frame during which the item functions flawlessly and without issue; this period of time is known as the grantee or warranty. The concept of reliability equivalency introduced, and applied to parallel systems with an exponential lifetime and independent, identical two-component series [2]. Three strategies identified for enhancing system reliability [3]. By utilizing these methods, one can enhance the reliability of a system by decreasing the failure rates by ρ, 0 < ρ < 1; warm duplication; perfect switch; and cold connected with a random switch. To evaluate the updated systems’ survival functions at various values for γ-fractiles in order to establish equality with the baseline. For a variety of systems, [4] examined the reliability equivalency factors of different Weibull failure rates. The reliability equivalency factors of exponentiated Weibull lifetimes distribution were identified [5]. Availability equivalency study conducted with the purpose of modeling a network of repairable bridges recently [6]. Engineering systems reliability evaluation was studied see [7]. Reliability engineering was introduced in [8]. Reliability equivalency in redundancy and reduction approaches was examined [9]. The dependability of a radar system based on Rayleigh distribution was improved [10]. The radar model’s reliability system was enhanced [11] using the two-parameter Weibull distribution as the basis. The Exponentiated Weibull Rayleigh Distribution was introduced and its properties were investigated [12]. The linear-exponential distribution function was assessed based on systems’ reliability [13]. Using a modified Weibull distribution, [14] the reliability of the engineering system was estimated. Alpha power transformation of the Sujatha distribution [15] used to examine the performance of a parallel-series system. The system reliability of the Standby redundancy approach was enhanced [16]. The generalized exponential lifetime model’s generic series-parallel system performance was improved [17]. Standby redundancy method used to assess the reliability of the system see [18]. The equivalency factor concept was calculated for a network with m non-identical mixed lifetimes [19]. A universal repairable parallel-series system’s availability equivalence factors were created [20]. To increase the structural reliability of the bridge, in [21] used a linear failure rate distribution. Exponentiated Weibull distribution was used [22] to evaluate the engineering system’s performance and dependability. Series systems with independent and non-identical components were found to be equivalent [23]. The Sine Half-Logistic Inverse Rayleigh Distribution was proposed, its properties were examined, and its applicability to biomedical Data was demonstrated [24]. This study investigates four strategies to increase the systems’ reliability: lowering the failure rate, using imperfect switch redundancy, warm redundancy, and cold redundancy. Abridge structure with identical and independent items with Rayleigh distribution lifetimes are used. The objective is to ascertain the optimal approach for augmenting the overall reliability of the system. The equivalency factors, γ–fractiles, and a simulation gave obvious evidence that the upgraded system performs better than the original one and the best strategy is by cold duplication.

## 2 Bridge Structure with two parameters Rayleigh distribution

Rayleigh distribution’s probability density function (PDF) and cumulative distribution function (CDF):

$$f(t,\alpha ,\beta )=\frac{\left(t-\alpha \right)}{\beta^{2}}exp\{-\frac{{\left(t-\alpha \right)}^{2}}{2\beta^{2}}\},t>\alpha ,\alpha ,\beta >0$$ (1)

$$F\left(t,\alpha ,\beta \right)=1-exp\left\{-\frac{{\left(t-\alpha \right)}^{2}}{2\beta^{2}}\right\}.$$ (2)

And its reliability function, hazard rate are given by:

$$R\left(t\right)=exp\left\{-\frac{{\left(t-\alpha \right)}^{2}}{2\beta^{2}}\right\},$$ (3)

$$h\left(t\right)=\frac{\left(t-\alpha \right)}{\beta^{2}},\;\alpha <t<\infty .$$ (4)

It is obvious from Eq (4) that the hazard rate is an increasing function of time t.

**Fig 1:**
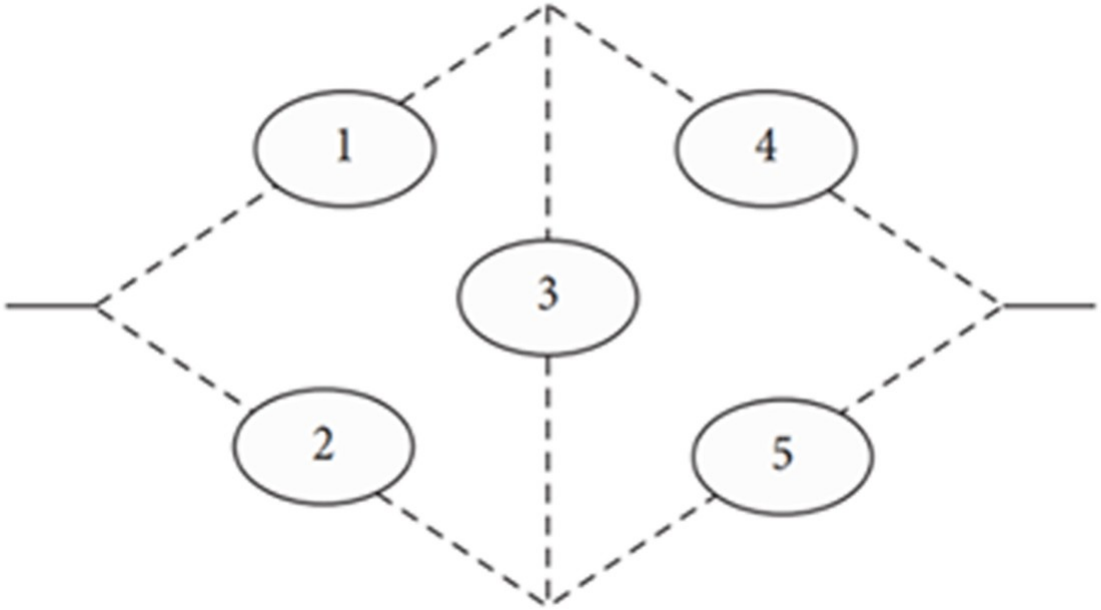
Coherent bridge system.

The bridge structure is considered as one of the advanced and complicated coherent system. Bridge network systems are vital infrastructures that need to be highly reliable in order to guarantee both functioning and safety. The five items of the Bridge structure are linked in series and parallel [8], as shown in Fig 1.

The component i, i = 1,2,…, 5, in this design is independent and identical with reliability function R_i_, i = 1,2,…, 5. The lifetimes and failure rates for each component is Rayleigh distribution and its reliability function is given by:

$${\text{R}}_{\text{i}}\left(\text{t}\right)=exp\left\{-\frac{{\left(t-\alpha_{i}\right)}^{2}}{2{\beta_{i}}^{2}}\right\}.$$ (5)

**Fig 2:**
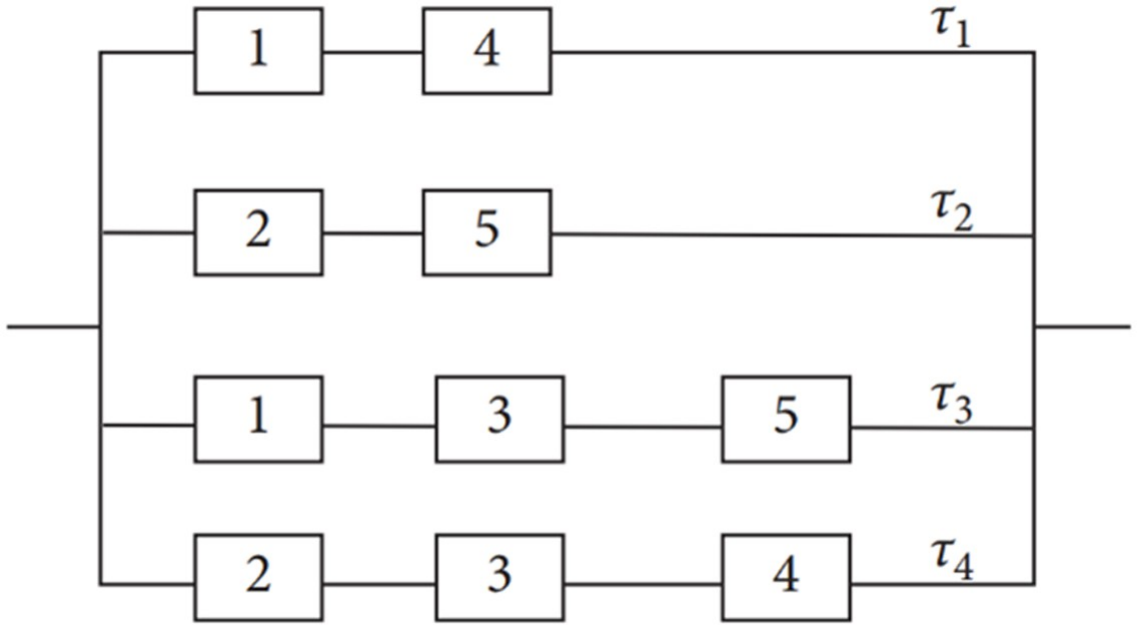
The equivalent design (Tie sets) for the bridge network system.

To construct the structure function for this system, some methods such as Tie Sets, can be used to assess the bridge network system’s reliability [7]. A Tie Set is the minimal path where, in our case the sets {1, 4}, {2, 5}, {1, 3, 5}, and {2, 3, 4} are the minimal paths. Since it is sufficient for the system to operate, all elements of at least one of the four sets shown above must operate. So the structural planning of this system can be redesigned as in Fig 2. The equivalent design consists of four parallel paths, where each path its elements are connected in series.

The reliability function (RF) of the Bridge calculated from the following equation:

$$\begin{array}{ll} \text{R}\left(\text{t}\right) & =\text{p}\left(\tau_{1}\cup \tau_{2}\cup \tau_{3}\cup \tau_{4}\right)\\ & =R_{1}R_{4}+R_{2}R_{5}+R_{2}R_{3}R_{4}+R_{1}R_{3}R_{5}-\overset{5}{\underset{i=1}{\sum }}\prod_{j\in N_{i}}R_{i}+2\prod_{j\in N_{i}}R_{i}. \end{array}$$ (6)

N = {1,2, …, 5}, N_i_ = N\{i}, i ∈ N. Considering that the items are identical in Eqs (5) and (6), we get:

$$\begin{array}{ll} R\left(t\right) & =exp\left\{-\frac{{\left(t-\alpha \right)}^{2}}{\beta^{2}}\right\}\{2+2\;exp\left\{-\frac{{\left(t-\alpha \right)}^{2}}{2\beta^{2}}\right\}-5\;exp\left\{-\frac{2{\left(t-\alpha \right)}^{2}}{\beta^{2}}\right\}\\ & +2\;exp\{-\frac{3{\left(t-\alpha \right)}^{2}}{2\beta^{2}}\}\}. \end{array}$$ (7)

(MTTF) mean time to failure can be calculated from the following equation:

$$MTTF=\int_{0}^{\infty }R\left(t\right)dt.$$ (8)

Eq (8) With the Mathematica Program system, it can be numerically calculated.

## 3 Methods of improved system

The Bridge system can be improved by improving some of its components according to one of the following four different methods:

1. Reduction method (RM): it is assumed that the failure rate of the system components is reducing by a factor ρ, 0 < ρ < 1.
2. Hot duplication method (HDM): this method assumes that the component is duplicated by a hot duplication component.
3. Cold duplication method (CDM): it is assumed the component is duplicated by a cold standby component.
4. Imperfect switch duplication method (ISDM): in this method it is assumed that a cold standby component is connected with the original component via an imperfect switch.

### 3.1 Reduction method

Assume the system will improve by decreasing the failure rates of the Bridge network design items in the set A using a factor ρ, 0 < ρ < 1. Assume that the improved system is reliability function as determined by the reduction method, is R_A,ρ_(t). For a component i ∈ R, R_A,ρ_(t) can be calculated as follows

(i) A ∈ S_1_ = {{3}}:
$$\begin{array}{ll} R_{A,\rho }\left(t\right) & =exp\left\{-\frac{{\left(t-\alpha \right)}^{2}}{\beta^{2}}\right\}[2-exp\left\{-\frac{{\left(t-\alpha \right)}^{2}}{\beta^{2}}\right\}\\ & +2\;exp\left\{-\rho \frac{{\left(t-\alpha \right)}^{2}}{2\beta^{2}}\right\}{\left(1-exp\left\{-\frac{{\left(t-\alpha \right)}^{2}}{2\beta^{2}}\right\}\right)}^{2}]. \end{array}$$ (9)
(ii) A ∈ S_2_ = {{1},{2},{4},{5}}:
$$\begin{array}{ll} R_{A,\rho }\left(t\right) & =exp\left\{-\frac{{\left(t-\alpha \right)}^{2}}{\beta^{2}}\right\}\left[1+exp\left\{-\frac{{\left(t-\alpha \right)}^{2}}{2\beta^{2}}\right\}-exp\left\{-\frac{{\left(t-\alpha \right)}^{2}}{\beta^{2}}\right\}\right]\\ & +exp\left\{-\left(1+\rho \right)\frac{{\left(t-\alpha \right)}^{2}}{2\beta^{2}}\right\}[1+exp\left\{-\frac{{\left(t-\alpha \right)}^{2}}{2\beta^{2}}\right\}-4\;exp\left\{-\frac{{\left(t-\alpha \right)}^{2}}{\beta^{2}}\right\}\\ & +2\;exp\left\{-\frac{3\;{\left(t-\alpha \right)}^{2}}{2\beta^{2}}\right\}]. \end{array}$$ (10)
(iii) A ∈ S_3_ = {{1,3},{2,3},{3,4},{3,5}}:
$$\begin{array}{ll} R_{A,\rho }\left(t\right) & =exp\left\{-\frac{{\left(t-\alpha \right)}^{2}}{\beta^{2}}\right\}+exp\left\{-\left(1+\rho \right)\frac{{\left(t-\alpha \right)}^{2}}{2\beta^{2}}\right\}[1+exp\left\{-\frac{{\left(t-\alpha \right)}^{2}}{2\beta^{2}}\right\}\\ & -2\;exp\left\{-\frac{{\left(t-\alpha \right)}^{2}}{\beta^{2}}\right\}\\ & +\;exp\left\{-\rho \frac{{\left(t-\alpha \right)}^{2}}{2\beta^{2}}\right\}\left(1-3\;exp\left\{-\frac{{\left(t-\alpha \right)}^{2}}{2\beta^{2}}\right\}+2\;exp\left\{-\frac{{\left(t-\alpha \right)}^{2}}{\beta^{2}}\right\}\right)]. \end{array}$$ (11)
(iv) A ∈ S_4_ = {{1,2},{4,5}}:
$$\begin{array}{ll} R_{A,\rho }\left(t\right) & =exp\left\{-\left(1+\rho \right)\frac{{\left(t-\alpha \right)}^{2}}{2\beta^{2}}\right\}[2\left[1+exp\left\{-\frac{{\left(t-\alpha \right)}^{2}}{2\beta^{2}}\right\}-exp\left\{-\frac{{\left(t-\alpha \right)}^{2}}{\beta^{2}}\right\}\right]\\ & +exp\left\{-\left(1+\rho \right)\frac{{\left(t-\alpha \right)}^{2}}{2\beta^{2}}\right\}\left(2\;exp\left\{-\frac{{\left(t-\alpha \right)}^{2}}{2\beta^{2}}\right\}-3\right)]. \end{array}$$ (12)
(v) A ∈ S_5_ = {{1,4},{2,5}}:
$$\begin{array}{ll} R_{A,\rho }\left(t\right) & =exp\left\{-\frac{{\left(t-\alpha \right)}^{2}}{\beta^{2}}\right\}+2\;exp\;\left\{-\left(1+\rho \right)\frac{{\left(t-\alpha \right)}^{2}}{2\beta^{2}}\right\}\left[1-exp\left\{-\frac{{\left(t-\alpha \right)}^{2}}{\beta^{2}}\right\}\right]\\ & +exp\left\{-\rho \frac{{\left(t-\alpha \right)}^{2}}{\beta^{2}}\right\}\left[1-\;3\;exp\left\{-\frac{{\left(t-\alpha \right)}^{2}}{\beta^{2}}\right\}+2\;exp\left\{-\frac{3{\left(t-\alpha \right)}^{2}}{2\beta^{2}}\right\}\right]. \end{array}$$ (13)

MTTF for the upgraded design via reduction method, which denoted by MTTFA,ρ, can be calculated by

$$MTTF_{A,\rho }=\int_{0}^{\infty }R_{A,\rho }\left(t\right)dt.$$ (14)

The integration in Eq (14) can be computed with the use of the Mathematica Program system. For each A ∈ S_i_, i = 1, 2,…, 6 and various values for α, β and ρ.

### 3.2 Hot duplication method

**Fig 3:**
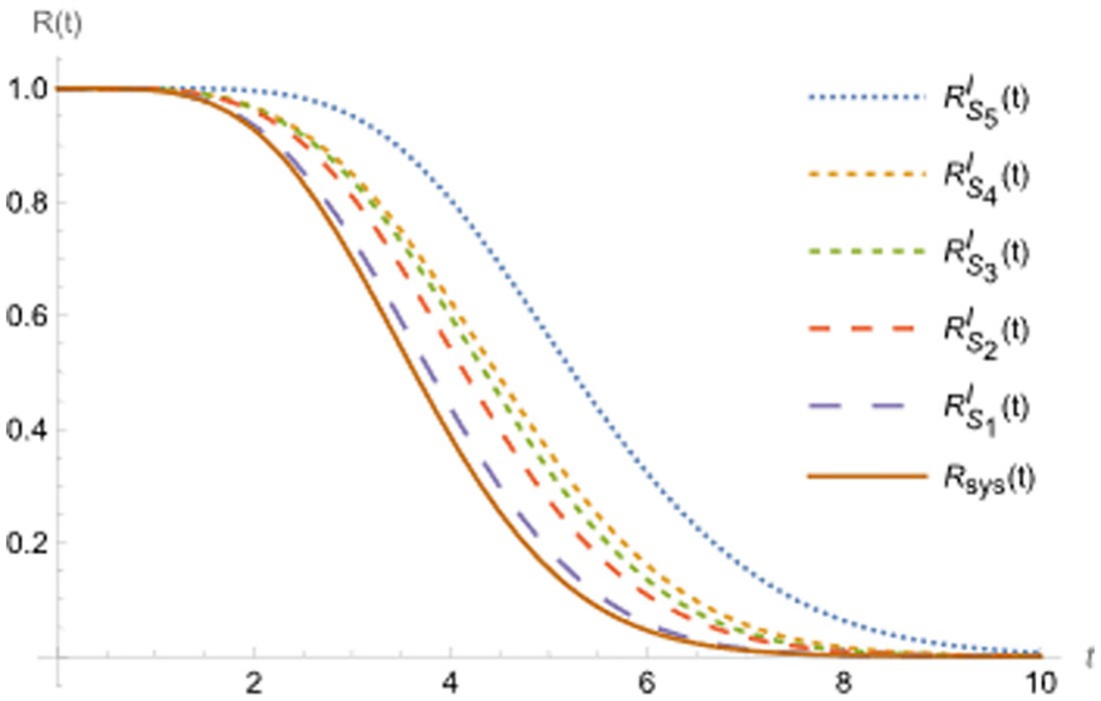
R(t) and $R_{B}^{D}\left(t\right)$ at B ∈ S_i_ and D = I.

In this paragraph, suppose that ${\text{R}}_{\text{B}}^{\text{H}}\left(\text{t}\right)$ denotes the system’s reliability belonging to the set B of items that are improved according to hot duplication improves HDM, as shown in Fig 3.

The item i ∈ B_i_, i = 1, 2,…, 6 has the reliability, say ${\text{R}}_{\text{i}}^{\text{H}}\left(\text{t}\right)$, as follows:

$$R_{i}^{H}\left(t\right)=\left(2-R_{i}\left(t\right)\right)R_{i}\left(t\right)=\left(2-exp\left\{-\frac{{\left(t-\alpha_{i}\right)}^{2}}{2{\beta_{i}}^{2}}\right\}\right)exp\left\{-\frac{{\left(t-\alpha_{i}\right)}^{2}}{2{\beta_{i}}^{2}}\right\}.$$ (15)

Thus, the system reliability *${\text{R}}_{\text{B}}^{\text{H}}\left(\text{t}\right)$* calculated from the following equations:

(i) B ∈ S_1_ = {{3}}:
$$\begin{array}{ll} R_{B}^{H}\left(t\right) & =exp\left\{-\frac{{\left(t-\alpha \right)}^{2}}{\beta^{2}}\right\}[2+4\;exp\left\{-\frac{{\left(t-\alpha \right)}^{2}}{2\beta^{2}}\right\}-11\;exp\left\{-\frac{{\left(t-\alpha \right)}^{2}}{\beta^{2}}\right\}+8\;exp\left\{-\frac{3{\left(t-\alpha \right)}^{2}}{2\beta^{2}}\right\}\\ & -2\;exp\left\{-\frac{2{\left(t-\alpha \right)}^{2}}{\beta^{2}}\right\}]. \end{array}$$ (16)
(ii) B ∈ S_2_ = {{1},{2},{4},{5}}:
$$\begin{array}{ll} R_{B}^{H}\left(t\right) & =exp\left\{-\frac{{\left(t-\alpha \right)}^{2}}{\beta^{2}}\right\}[3+2\;exp\left\{-\frac{{\left(t-\alpha \right)}^{2}}{2\beta^{2}}\right\}-10\;exp\left\{-\frac{{\left(t-\alpha \right)}^{2}}{\beta^{2}}\right\}+8\;exp\left\{-\frac{3{\left(t-\alpha \right)}^{2}}{2\beta^{2}}\right\}\\ & -2\;exp\left\{-\frac{2{\left(t-\alpha \right)}^{2}}{\beta^{2}}\right\}]. \end{array}$$ (17)
(iii) B ∈ S_3_ = {{1,3},{2,3},{3,4},{3,5}}:
$$\begin{array}{ll} R_{B}^{H}\left(t\right) & =exp\left\{-\frac{{\left(t-\alpha \right)}^{2}}{\beta^{2}}\right\}[3+5\;exp\left\{-\frac{{\left(t-\alpha \right)}^{2}}{2\beta^{2}}\right\}-21\;exp\left\{-\frac{{\left(t-\alpha \right)}^{2}}{\beta^{2}}\right\}\\ & +23\;exp\left\{-\frac{3{\left(t-\alpha \right)}^{2}}{2\beta^{2}}\right\}-11\;exp\left\{-\frac{2{\left(t-\alpha \right)}^{2}}{\beta^{2}}\right\}+2\;exp\left\{-\frac{5{\left(t-\alpha \right)}^{2}}{2\beta^{2}}\right\}]. \end{array}$$ (18)
(iv) B ∈ S_4_ = {{1,2},{4,5}}:
$$\begin{array}{ll} R_{B}^{H}\left(t\right) & =exp\left\{-\frac{{\left(t-\alpha \right)}^{2}}{\beta^{2}}\right\}[4+2\;exp\left\{-\frac{{\left(t-\alpha \right)}^{2}}{2\beta^{2}}\right\}-18\;exp\left\{-\frac{{\left(t-\alpha \right)}^{2}}{\beta^{2}}\right\}\\ & +22\;exp\left\{-\frac{3{\left(t-\alpha \right)}^{2}}{2\beta^{2}}\right\}-11\;exp\left\{-\frac{2{\left(t-\alpha \right)}^{2}}{\beta^{2}}\right\}+2\;exp\left\{-\frac{5{\left(t-\alpha \right)}^{2}}{2\beta^{2}}\right\}]. \end{array}$$ (19)
(v) B ∈ S_5_ = {{1,4},{2,5}}:
$$\begin{array}{ll} R_{B}^{H}\left(t\right) & =exp\left\{-\frac{{\left(t-\alpha \right)}^{2}}{\beta^{2}}\right\}[5-17\;exp\left\{-\frac{{\left(t-\alpha \right)}^{2}}{\beta^{2}}\right\}+22\;exp\left\{-\frac{3{\left(t-\alpha \right)}^{2}}{2\beta^{2}}\right\}\\ & -11\;exp\left\{-\frac{2{\left(t-\alpha \right)}^{2}}{\beta^{2}}\right\}+2\;exp\left\{-\frac{5{\left(t-\alpha \right)}^{2}}{2\beta^{2}}\right\}]. \end{array}$$ (20)

MTTF for the upgraded design via warm duplication method, which denoted by ${MTTF}_{B}^{H}$, can be calculated by

$$MTTF_{B}^{H}=\int_{0}^{\infty }R_{B}^{H}\left(t\right)dt.$$ (21)

There is no theoretical solution to Eq (21), hence some numerical programs must be used to calculate the values of α and β, for each B ∈ S_i_, i = 1, 2,…, 1.

### 3.3 Cold duplication method

**Fig 4:**
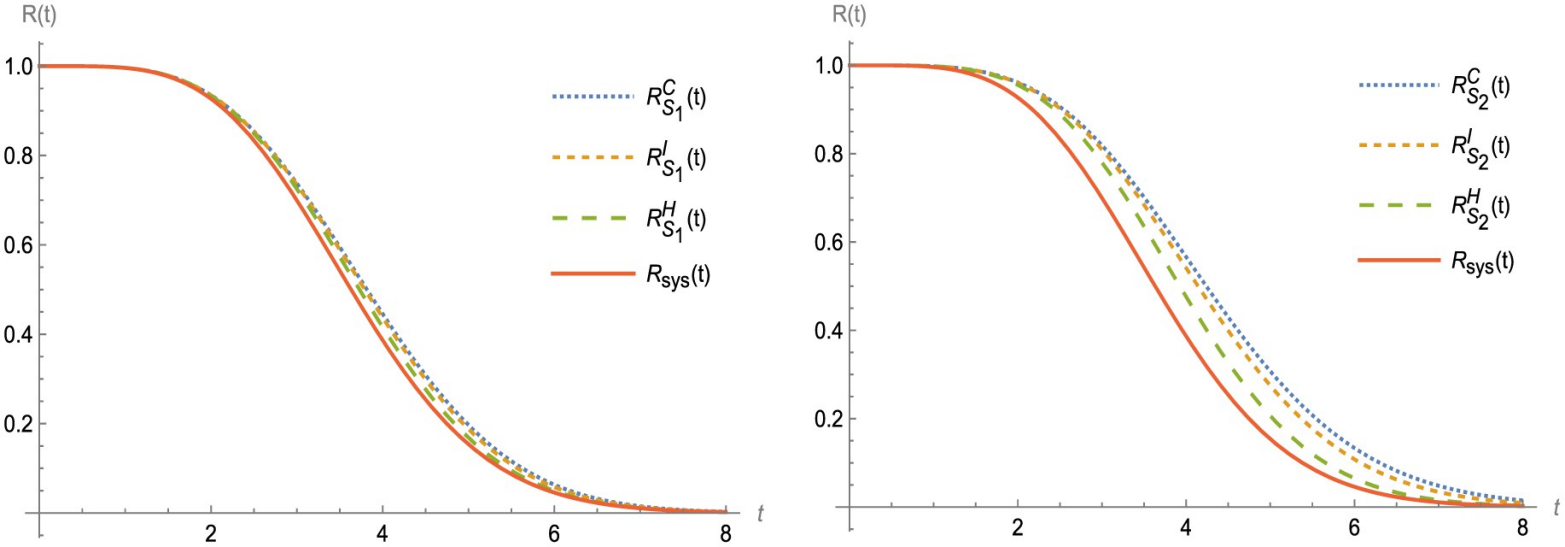
R(t) and $R_{B}^{D}\left(t\right)$, at B ∈ S_1_ and S_2_.

In this section, suppose that $R_{B}^{C}(t)$ refers to the reliability of the upgraded design obtained via cold duplication, where a perfect switch connects a component from set B with a component from an identical set. $R_{i}^{C}(t)$ can be used to compute the reliability of the item i ∈ B_i_, i = 1, 2,…, 6 as illustrated in Fig 4.

$$\begin{array}{ll} R_{i}^{C}\left(t\right) & =R_{i}\left(t\right)+\overset{t}{\underset{0}{\int }}f_{i}\left(x\right)R_{i}\left(t-x\right)dx\\ & =exp\left\{-{\left(\frac{t}{\alpha_{i}}\right)}^{\beta_{i}}\right\}\\ & +\overset{t}{\underset{0}{\int }}\frac{\beta_{i}}{\alpha_{i}}{\left(\frac{x}{\alpha_{i}}\right)}^{\beta_{i}-1}exp\left\{-{\left(\frac{x}{\alpha_{i}}\right)}^{\beta_{i}}\right\}\;exp\left\{-{\left(\frac{t-x}{\alpha_{i}}\right)}^{\beta_{i}}\right\}dx. \end{array}$$ (22)

Thus, the system reliability $R_{B}^{C}(t)$ calculated from the following equations:

(i) B ∈ S_1_ = {{3}}:
$$\begin{array}{ll} R_{B}^{C}\left(t\right) & =exp\left\{-\frac{{\left(t-\alpha \right)}^{2}}{\beta^{2}}\right\}[2-exp\left\{-\frac{{\left(t-\alpha \right)}^{2}}{\beta^{2}}\right\}\\ & +R_{B_{1}}^{C}\left(t\right)(2-4\;exp\left\{-\frac{{\left(t-\alpha \right)}^{2}}{2\beta^{2}}\right\}\\ & +2\;exp\left\{-\frac{{\left(t-\alpha \right)}^{2}}{\beta^{2}}\right\})]. \end{array}$$ (23)
(ii) B ∈ S_2_ = {{1},{2},{4},{5}}:
$$\begin{array}{ll} R_{B}^{C}\left(t\right) & =exp\left\{-\frac{{\left(t-\alpha \right)}^{2}}{\beta^{2}}\right\}\left[1+exp\left\{-\frac{{\left(t-\alpha \right)}^{2}}{2\beta^{2}}\right\}-exp\left\{-\frac{{\left(t-\alpha \right)}^{2}}{\beta^{2}}\right\}\right]\\ & +R_{B_{2}}^{C}\left(t\right)exp\left\{-\frac{{\left(t-\alpha \right)}^{2}}{2\beta^{2}}\right\}[1+\;exp\left\{-\frac{{\left(t-\alpha \right)}^{2}}{2\beta^{2}}\right\}-4\;exp\left\{-\frac{{\left(t-\alpha \right)}^{2}}{\beta^{2}}\right\}\\ & +2\;exp\left\{-\frac{3{\left(t-\alpha \right)}^{2}}{2\beta^{2}}\right\}]. \end{array}$$ (24)
(iii) B ∈ S_3_ = {{1,3},{2,3},{3,4},{3,5}}:
$$\begin{array}{ll} R_{B}^{C}\left(t\right) & =exp\left\{-\frac{{\left(t-\alpha \right)}^{2}}{\beta^{2}}\right\}+R_{B_{3}}^{C}\left(t\right)exp\left\{-\frac{{\left(t-\alpha \right)}^{2}}{2\beta^{2}}\right\}[1+exp\left\{-\frac{{\left(t-\alpha \right)}^{2}}{2\beta^{2}}\right\}-2\;exp\left\{-\frac{{\left(t-\alpha \right)}^{2}}{\beta^{2}}\right\}\\ & +\;R_{B_{3}}^{C}\left(t\right)\left[1-3\;exp\left\{-\frac{{\left(t-\alpha \right)}^{2}}{2\beta^{2}}\right\}+2\;exp\left\{-\frac{{\left(t-\alpha \right)}^{2}}{2\beta^{2}}\right\}\right]]. \end{array}$$ (25)
(iv) B ∈ S_4_ = {{1,2},{4,5}}:
$$\begin{array}{ll} R_{B}^{C}\left(t\right) & =R_{B_{4}}^{C}\left(t\right)exp\left\{-\frac{{\left(t-\alpha \right)}^{2}}{2\beta^{2}}\right\}[2+2\;exp\left\{-\frac{{\left(t-\alpha \right)}^{2}}{2\beta^{2}}\right\}-2\;exp\left\{-\frac{{\left(t-\alpha \right)}^{2}}{\beta^{2}}\right\}\\ & +R_{B_{4}}^{C}\left(t\right)exp\left\{-\frac{{\left(t-\alpha \right)}^{2}}{2\beta^{2}}\right\}\left[-3+2\;exp\left\{-\frac{{\left(t-\alpha \right)}^{2}}{2\beta^{2}}\right\}\right]]. \end{array}$$ (26)
(v) B ∈ S_5_ = {{1,4},{2,5}}:
$$\begin{array}{ll} R_{B}^{C}\left(t\right) & =exp\left\{-\frac{{\left(t-\alpha \right)}^{2}}{\beta^{2}}\right\}+R_{B_{6}}^{C}\left(t\right)[2\;exp\left\{-\frac{{\left(t-\alpha \right)}^{2}}{\beta^{2}}\right\}-2\;exp\left\{-\frac{3{\left(t-\alpha \right)}^{2}}{2\beta^{2}}\right\}\\ & +R_{B_{6}}^{C}\left(t\right)\left[1-\;3\;exp\left\{-\frac{{\left(t-\alpha \right)}^{2}}{\beta^{2}}\right\}+2\;exp\left\{-\frac{3{\left(t-\alpha \right)}^{2}}{2\beta^{2}}\right\}\right]]. \end{array}$$ (27)

MTTF for the upgraded design by using cold duplication method, which denoted by ${MTTF}_{B}^{C}$, can be calculated by

$$MTTF_{B}^{C}=\int_{0}^{\infty }R_{B}^{C}\left(t\right)dt.$$ (28)

There is no theoretical solution to Eq (28), hence some numerical programs must be used to calculate the values of α and β, for each B ∈ S_i_, i = 1, 2,…, 5.

### 3.4 Imperfect switch duplication method (ISDM)

In this technique, the system will improve by upgrading the design, i.e., the item i, is linked to a cold redundant standby item via an imperfect switch with Rayleigh distribution and parameters,α, λ_s_. And the reliability function (RF) is given by:

$$R_{sw}\left(t\right)=exp\left\{-{\left(\frac{t-\alpha }{\lambda_{s}}\right)}^{2}\right\}.$$ (29)

We denoted by $R_{B}^{I}(t)$ to the reliability function of the upgraded design via imperfect switch, $R_{i}^{I}(t)$ calculated for the item i ∈ B_i_, i = 1, 2,…, 5, and the upgraded design by (ISDM) can be calculated from:

$$\begin{array}{ll} R_{i}^{I}\left(t\right) & =R_{i}\left(t\right)+\overset{t}{\underset{0}{\int }}f_{i}\left(x\right)R_{sw}\left(t-x\right)R_{i}\left(t-x\right)dx\\ & =exp\left\{-{\left(\frac{t}{\alpha_{i}}\right)}^{\beta_{i}}\right\}\\ & +\overset{t}{\underset{0}{\int }}\frac{\beta }{\alpha_{i}}{\left(\frac{x}{\alpha_{i}}\right)}^{\beta_{i}-1}exp\left\{-{\left(\frac{x}{\alpha_{i}}\right)}^{\beta_{i}}\right\}\;exp\left\{-{\left(\frac{t-x}{\alpha_{i}}\right)}^{\lambda_{s}}\right\}exp\left\{-{\left(\frac{t-x}{\alpha_{i}}\right)}^{\beta_{i}}\right\}dx. \end{array}$$ (30)

Thus, $R_{B}^{c}(t)$ calculated from the following equations:

(i) B ∈ S_1_ = {{3}}:
$$\begin{array}{ll} R_{B}^{I}\left(t\right) & =exp\left\{-\frac{{\left(t-\alpha \right)}^{2}}{\beta^{2}}\right\}[2-exp\left\{-\frac{{\left(t-\alpha \right)}^{2}}{\beta^{2}}\right\}\\ & +R_{B_{1}}^{I}\left(t\right)\left(2-4\;exp\left\{-\frac{{\left(t-\alpha \right)}^{2}}{2\beta^{2}}\right\}+2\;exp\left\{-\frac{{\left(t-\alpha \right)}^{2}}{\beta^{2}}\right\}\right)]. \end{array}$$ (31)
(ii) B ∈ S_2_ = {{1},{2},{4},{5}}:
$$\begin{array}{ll} R_{B}^{I}\left(t\right) & =exp\left\{-\frac{{\left(t-\alpha \right)}^{2}}{\beta^{2}}\right\}\left[1+exp\left\{-\frac{{\left(t-\alpha \right)}^{2}}{2\beta^{2}}\right\}-exp\left\{-\frac{{\left(t-\alpha \right)}^{2}}{\beta^{2}}\right\}\right]\\ & +R_{B_{2}}^{I}\left(t\right)exp\left\{-\frac{{\left(t-\alpha \right)}^{2}}{2\beta^{2}}\right\}[1+exp\left\{-\frac{{\left(t-\alpha \right)}^{2}}{2\beta^{2}}\right\}-4\;exp\left\{-\frac{{\left(t-\alpha \right)}^{2}}{\beta^{2}}\right\}\\ & +2\;exp\left\{-\frac{3{\left(t-\alpha \right)}^{2}}{2\beta^{2}}\right\}]. \end{array}$$ (32)
(iii) B ∈ S_3_ = {{1,3},{2,3},{3,4},{3,5}}:
$$\begin{array}{ll} R_{B}^{I}\left(t\right) & =exp\left\{-\frac{{\left(t-\alpha \right)}^{2}}{\beta^{2}}\right\}+R_{B_{3}}^{I}\left(t\right)exp\left\{-\frac{{\left(t-\alpha \right)}^{2}}{2\beta^{2}}\right\}[1+exp\left\{-\frac{{\left(t-\alpha \right)}^{2}}{2\beta^{2}}\right\}-2\;exp\left\{-\frac{{\left(t-\alpha \right)}^{2}}{\beta^{2}}\right\}\\ & +\;R_{3}^{I}\left(t\right)\left[1-3\;exp\left\{-\frac{{\left(t-\alpha \right)}^{2}}{2\beta^{2}}\right\}+2\;exp\left\{-\frac{{\left(t-\alpha \right)}^{2}}{\beta^{2}}\right\}\right]]. \end{array}$$ (33)
(iv) B ∈ S_4_ = {{1,2},{4,5}}:
$$\begin{array}{ll} R_{B}^{I}\left(t\right) & =R_{B_{4}}^{C}\left(t\right)exp\left\{-\frac{{\left(t-\alpha \right)}^{2}}{2\beta^{2}}\right\}[2+2\;exp\left\{-\frac{{\left(t-\alpha \right)}^{2}}{2\beta^{2}}\right\}-2\;exp\left\{-\frac{{\left(t-\alpha \right)}^{2}}{\beta^{2}}\right\}\\ & +R_{B_{4}}^{I}\left(t\right)exp\left\{-\frac{{\left(t-\alpha \right)}^{2}}{2\beta^{2}}\right\}\left[-3+2\;exp\left\{-\frac{{\left(t-\alpha \right)}^{2}}{2\beta^{2}}\right\}\right]]. \end{array}$$ (34)
(v) B ∈ S_5_ = {{1,4},{2,5}}:
$$\begin{array}{ll} R_{B}^{I}\left(t\right) & =exp\left\{-\frac{{\left(t-\alpha \right)}^{2}}{\beta^{2}}\right\}+R_{B_{6}}^{I}\left(t\right)[2\;exp\left\{-\frac{{\left(t-\alpha \right)}^{2}}{\beta^{2}}\right\}-2\;exp\left\{-\frac{3{\left(t-\alpha \right)}^{2}}{2\beta^{2}}\right\}\\ & +R_{B_{6}}^{I}\left(t\right)\left[1-\;3\;exp\left\{-\frac{{\left(t-\alpha \right)}^{2}}{\beta^{2}}\right\}+2\;exp\left\{-\frac{3{\left(t-\alpha \right)}^{2}}{2\beta^{2}}\right\}\right]]. \end{array}$$ (35)

MTTF for the upgraded design by using imperfect switch duplication method, which denoted by ${MTTF}_{B}^{I}$, can be calculated by

$$MTTF_{B}^{I}=\int_{0}^{\infty }R_{B}^{I}\left(t\right)dt.$$ (36)

There is no theoretical solution to Eq (36), hence some numerical programs must be used to calculate the values of α, β and λ_s_, for each B ∈ S_i_, i = 1, 2,…, 5.

## 4 γ-fractiles

In this section, we introduce the *γ*-fractiles of the original and the upgraded systems. Let *L*(*γ*) and $L_{B}^{D}(\gamma )$ denote the *γ* -fractile of the original and upgraded systems. For both the beginning and the upgraded designs are calculated as the crucial indicator of reliability system performance. The following equations with respect to L can obtain the γ-fractiles.


$$R\left(L\left(\gamma \right)\right)=\gamma ,\;\;R_{B}^{D}\left(L_{B}^{D}\left(\gamma \right)\right)=\gamma ,\;\;\;\;\;\;D=H,I\;and\;C.$$ (37)

By substituting from (7) into the 1^st^ part of (37), to create the γ -fractiles for the beginning system, the following equation can be solved in L.


$$exp\left\{-2\frac{{\left(L-\alpha \right)}^{2}}{2\beta^{2}}\right\}\left[2+2\;exp\left\{-\frac{{\left(L-\alpha \right)}^{2}}{2\beta^{2}}\right\}-5\;exp\left\{-2\frac{{\left(L-\alpha \right)}^{2}}{2\beta^{2}}\right\}+2\;exp\left\{-3\frac{{\left(L-\alpha \right)}^{2}}{2\beta^{2}}\right\}\right]=\gamma .$$ (38)

Similarity, by replacing from (16–20), (31–35) and (23–27) into the 2^nd^ part of (37), γ-fractiles for (HDM, ISDM and CDM), and $L_{B}^{D}\left(\gamma \right)$, D = H, I, C, respectively, are obtained.

Because the resulting equations for γ -fractiles have no explicit formula for the solution, so we used numerical formula, a code in Mathematica (12.3) application, and we have used an Intel(R) Core (TM) I7 PC with 3.00 GHz.

## 5 Reliability equivalence factors

In this section, A bridge network system’s (REF) is described. The REF should be used for reducing component failure rates so that the better system, is known.

The REF ${(\rho }_{A,B}^{D}\left(\gamma \right))$, for sets A and B have been improved via reduction and duplication techniques, respectively. Eq (39) solved for ρ, to get the factor $\rho_{A,B}^{D}\left(\gamma \right)$.


$$R_{B}^{D}\left(t\right)=R_{A,\rho }\left(t\right)=\gamma ,D=H,I\;and\;C.$$ (39)

And A, B ∈ S_i_, i = 1, 2,…, 5.

- Substituting from (9)–(13) and (16)–(20) in (39), when D = H, the hot REFs, $\rho_{A,B}^{H}\left(\gamma \right)$, can be calculated.
- Also, replacing from (9)–(13), (31)–(35) in (39), at D = I, $\rho_{A,B}^{I}\left(\gamma \right)$ can be calculated.
- The cold REFs, $\rho_{A,B}^{C}\left(\gamma \right)$, can be calculated by replacing from (9)–(13), (23)–(27) into (39), at D = C.

Since $\rho_{A,B}^{D}\left(\gamma \right)$ system of equations has not a closed form solution, so used numerical code.

## 6 Simulation example

In this paragraph, we derived a simulation example, to illustrate the theoretical results in the above sections. Consider the following assumptions:

1. Each component of the system is independent and identical.
2. Each component has two parameters Weibull distribution, α = 0.1 and β = 3.
3. In the ISDM, the parameters for the switch are α = 0.1 and β = 3 and λ_s_ = 2.7.

For this example, MTTF = 3.6221, and Table 1 shows the ${MTTF}_{B}^{D}$ for the upgraded design for all subsets of B.

**Table 1:** ${MTTF}_{B}^{D}$, D = H, C; B ∈ S_i_, i = 1, 2,…, 6.

B ∈ S_i_	${MTTF}_{B}^{H}$	${MTTF}_{B}^{I}$	${MTTF}_{B}^{C}$
S_1_	3.70289	3.76488	3.79252
S_2_	3.89994	4.13667	4.25267
S_3_	3.98246	4.31659	4.49928
S_4_	4.08715	4.43658	4.59676
S_5_	4.28419	5.28291	6.19615

From Table 1, we can conclude that:

$MTTF<{MTTF}_{B}^{H}\;<\;{MTTF}_{B}^{I}\;<{\;MTTF}_{B}^{C}$, for all B ∈ S_i_, in all studied cases.

$MTTF<{MTTF}_{S_{1}}^{D}<{MTTF}_{S_{2}}^{D}<{\;MTTF}_{S_{3}}^{D}<\;{MTTF}_{S_{4}}^{D}\;<\;{MTTF}_{S_{5}}^{D}$, D = H, I, C.

The L(γ), $L_{B}^{D}\left(\gamma \right)$ and $\rho_{A,B}^{D}\left(\gamma \right)$, are computed with code by the wolfram Mathematic, at D = H, I, C; A, B ∈ S_i_, i = 1, 2,…, 5. The values of γ are: 0.1, 0.2,⋯, 0.9.

**Fig 5:**
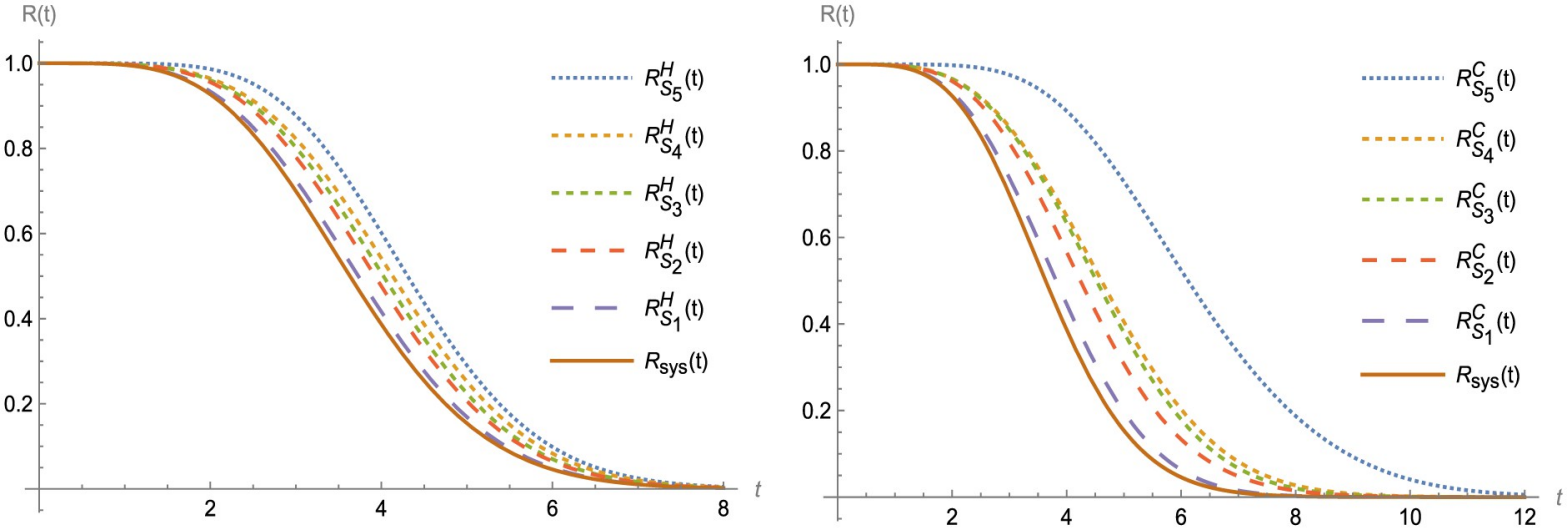
R(t) and $R_{B}^{D}\left(t\right)$, at B ∈ S_i_ and D = H and C.

**Fig 6:**
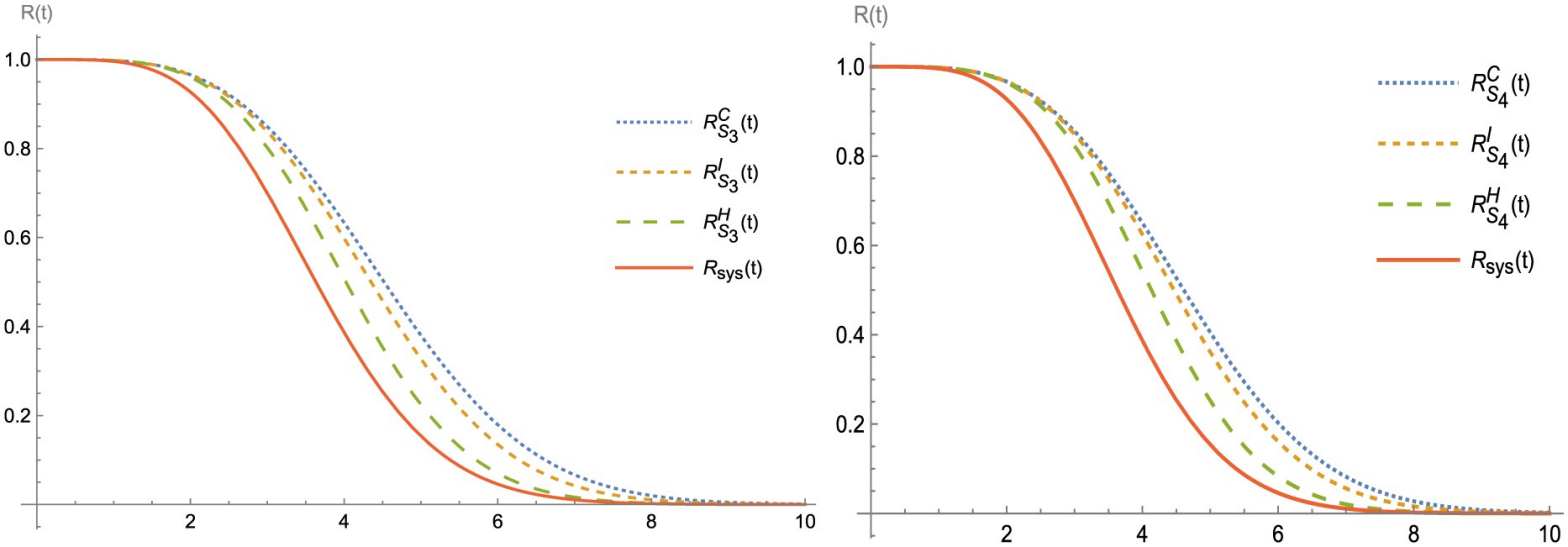
R(t) and $R_{B}^{D}\left(t\right)$, at B ∈ S_3_ and S_4_.

**Fig 7:**
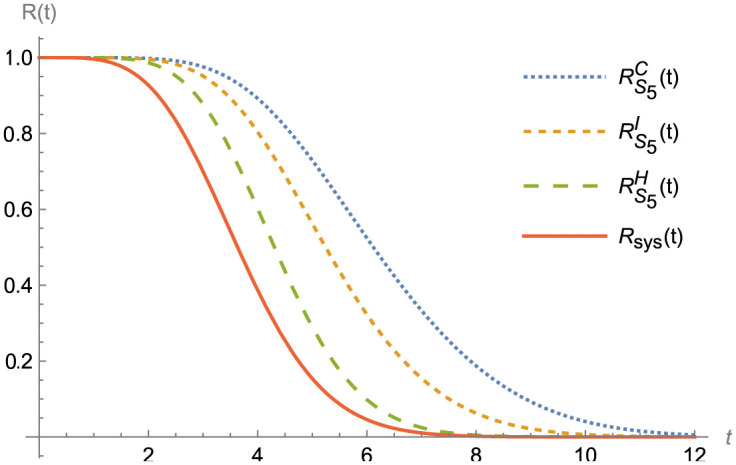
R(t) and $R_{B}^{D}\left(t\right)$, at B ∈ S_5_.

Figs 3–7 shows (RF) for the beginning and upgraded design for some subsets of B ∈ S_i_, i = 1, 2,…, 5, and Tables 2 and 3, conclude that:

1. $R\left(t\right)<R_{B}^{H}\left(t\right)<R_{B}^{I}\left(t\right)<R_{B}^{C}\left(t\right)$, at B ∈ S_i_, i = 1, 2,…, 5.
2. $R\left(t\right)<R_{S_{1}}^{D}(t)<R_{S_{2}}^{D}(t)<{\;R}_{S_{3}}^{D}\left(t\right)<\;R_{S_{4}}^{D}\;\left(t\right)<\;R_{S_{5}}^{D}\left(t\right),$ at D = H, C and I.
3. $L\left(\gamma \right)<L_{B}^{H}\left(\gamma \right)<L_{B}^{I}\left(\gamma \right)<L_{B}^{C}\left(\gamma \right)$, at B ∈ S_i_, i = 1, 2,…, 5.
4. $L\left(\gamma \right)<L_{S_{1}}^{D}\left(\gamma \right)<L_{S_{2}}^{D}(\gamma )<L_{S_{3}}^{D}(\gamma )<L_{S_{4}}^{D}(\gamma )<L_{S_{5}}^{D}(\gamma )$, at D = H, I and C.

**Table 2:** L(γ), $L_{B}^{D}(\gamma )$, D = H, I; B ∈ S_i_, i = 1, 2,…, 5.

γ	L	Hot, B ∈ S_i_					Imperfect switch, B ∈ S_i_				
		S_1_	S_2_	S_3_	S_4_	S_5_	S_1_	S_2_	S_3_	S_4_	S_5_
0.1	5.3815	5.4559	5.6542	5.7208	5.8509	5.9868	5.5695	6.0714	6.2776	6.4779	7.5049
0.2	4.7470	4.8359	5.0298	5.1121	5.2344	5.3785	4.9363	5.3693	5.5908	5.7586	6.6741
0.3	4.3121	4.4076	4.6002	4.6913	4.8081	4.9609	4.4949	4.8898	5.1129	5.2595	6.1094
0.4	3.9547	4.0531	4.2459	4.3421	4.4545	4.6173	4.1276	4.4964	4.7147	4.8445	5.6514
0.5	3.6322	3.7304	3.9247	4.0235	4.1318	4.3066	3.7926	4.1414	4.3504	4.4654	5.2436
0.6	3.3208	3.4160	3.6126	3.7116	3.8155	4.0056	3.4659	3.7978	3.9932	4.0941	4.8547
0.7	2.9997	3.0887	3.2885	3.3847	3.4832	3.6939	3.1264	3.4427	3.6191	3.7051	4.4586
0.8	2.6403	2.7184	2.9219	3.0109	3.1015	3.3424	2.7436	3.0428	3.1926	3.2613	4.0195
0.9	2.1726	2.2313	2.4365	2.5091	2.5844	2.8786	2.2436	2.5176	2.6259	2.6716	3.4510

**Table 3:** L(γ), $L_{B}^{D}(\gamma )$, D = C; B ∈ S_i_, i = 1, 2,…, 5.

γ	L	Cold, B ∈ S_i_				
		S_1_	S_2_	S_3_	S_4_	S_5_
0.1	5.3815	5.6274	6.2993	6.6161	6.7975	8.9030
0.2	4.7470	4.9831	5.5444	5.8703	6.0159	7.8948
0.3	4.3121	4.5330	5.0324	5.3503	5.4726	7.1977
0.4	3.9547	4.1584	4.6147	4.9168	5.0210	6.6273
0.5	3.6322	3.8169	4.2394	4.5205	4.6089	6.1187
0.6	3.3208	3.4844	3.8779	4.1328	4.2065	5.6359
0.7	2.9997	3.1394	3.5057	3.7283	3.7874	5.1484
0.8	2.6403	2.7516	3.0889	3.2702	3.3140	4.6151
0.9	2.1726	2.2471	2.5452	2.6691	2.6952	3.9368

Tables 4 and 5 provide the numerical values of REFs for some sets A, B ∈ S_i_, i = 1, 2,…, 5.

**Table 4:** $\rho_{A,B}^{D}$, when D = H, I, at A, B ∈ S_i_, i = 1, 2,…, 5.

γ	A ∈ S_i_	Hot, B ∈ S_i_					Imperfect Switch, B ∈ S_i_				
		S_1_	S_2_	S_3_	S_4_	S_5_	S_1_	S_2_	S_3_	S_4_	S_5_
0.1	S_1_	0.6323	0.1102	−	−	−	0.2884	−	−	−	−
	S_2_	0.8883	0.6506	0.5851	0.4724	0.3716	0.7432	0.3159	0.1979	0.1020	−
	S_3_	0.9096	0.7120	0.6566	0.5606	0.4745	0.7898	0.4270	0.3267	0.2460	−
	S_4_	0.9414	0.7966	0.7515	0.8779	0.5866	0.8565	0.5389	0.4311	0.3367	−
	S_5_	0.9438	0.8225	0.7889	0.7309	0.6793	0.8699	0.6509	0.5909	0.5428	0.3828
0.2	S_1_	0.5683	0.0370	−	−	−	0.2503	−	−	−	−
	S_2_	0.8489	0.5894	0.5004	0.3852	0.2700	0.7044	0.2767	0.1305	0.0395	−
	S_3_	0.8817	0.6716	0.5981	0.5021	0.4058	0.7657	0.4114	0.2896	0.2145	−
	S_4_	0.9201	0.7602	0.6978	0.8758	0.5155	0.8349	0.5213	0.3889	0.2990	−
	S_5_	0.9238	0.7905	0.7443	0.6845	0.6248	0.8498	0.6283	0.5531	0.5068	0.3462
0.3	S_1_	0.5182	−	−	−	−	0.2219	−	−	−	−
	S_2_	0.8212	0.5413	0.4359	0.3190	0.1908	0.6832	0.2474	0.0843	−	−
	S_3_	0.8622	0.6382	0.5519	0.4555	0.3493	0.7528	0.3962	0.2613	0.1898	−
	S_4_	0.9051	0.7309	0.6557	0.8712	0.4564	0.8234	0.5057	0.3575	0.2702	−
	S_5_	0.9097	0.7652	0.7102	0.6490	0.5820	0.8388	0.6116	0.5267	0.4817	0.3179
0.4	S_1_	0.4729	−	−	−	−	0.1972	−	−	−	−
	S_2_	0.7996	0.4979	0.3797	0.2615	0.1197	0.6707	0.2220	0.0475	−	−
	S_3_	0.8466	0.6065	0.5100	0.4127	0.2955	0.7452	0.3802	0.2362	0.1676	−
	S_4_	0.8932	0.7037	0.6177	0.5239	0.3993	0.8169	0.4906	0.3303	0.2446	−
	S_5_	0.8986	0.7424	0.6804	0.5892	0.5433	0.8324	0.5973	0.5055	0.4618	0.2930
0.5	S_1_	0.4289	−	−	−	−	0.1740	−	−	−	−
	S_2_	0.7822	0.4557	0.3273	0.2078	0.0504	0.6647	0.1981	0.0164	−	−
	S_3_	0.8336	0.5738	0.4690	0.3705	0.2403	0.7415	0.3625	0.2123	0.1461	−
	S_4_	0.8838	0.6764	0.5809	0.4832	0.3388	0.8143	0.4749	0.3049	0.2199	−
	S_5_	0.8898	0.7203	0.6525	0.6181	0.5056	0.8293	0.5840	0.4876	0.4450	0.2692
0.6	S_1_	0.3837	−	−	−	−	0.1511	−	−	−	−
	S_2_	0.7689	0.4122	0.2759	0.1553	0.0210	0.6650	0.1743	−	−	−
	S_3_	0.8230	0.5382	0.4263	0.3262	0.1796	0.7414	0.3421	0.1879	0.1242	−
	S_4_	0.8765	0.6473	0.5431	0.4408	0.2690	0.8152	0.4576	0.2796	0.1949	−
	S_5_	0.8830	0.6975	0.6252	0.5609	0.4666	0.8295	0.5711	0.4719	0.4307	0.2452
0.7	S_1_	0.3347	−	−	−	−	0.1272	−	−	−	−
	S_2_	0.7605	0.3648	0.2234	0.1019	−	0.6727	0.1494	−	−	−
	S_3_	0.8152	0.4965	0.3791	0.2774	0.1084	0.7459	0.3172	0.1618	0.1009	−
	S_4_	0.8720	0.6141	0.5019	0.3936	0.1787	0.8205	0.4375	0.2528	0.1679	−
	S_5_	0.8788	0.6728	0.5975	0.5324	0.4238	0.8337	0.5578	0.4582	0.4189	0.2195
0.8	S_1_	0.2776	−	−	−	−	0.1008	−	−	−	−
	S_2_	0.7601	0.3091	0.1666	0.0454	−	0.6922	0.1214	−	−	−
	S_3_	0.8120	0.4431	0.3228	0.2195	0.0159	0.7577	0.2843	0.1318	0.0748	−
	S_4_	−	0.5725	0.4532	0.3366	0.0324	0.8325	0.4116	0.2224	0.1366	−
	S_5_	0.8787	0.6439	0.5679	0.5026	0.3729	0.8439	0.5436	0.4470	0.4106	0.1897
0.9	S_1_	0.2022	−	−	−	−	0.0684	−	−	−	−
	S_2_	0.7788	0.2337	0.1001	−	−	0.7369	0.0858	−	−	−
	S_3_	0.8210	0.3615	0.2450	0.1426	−	0.7866	0.2324	0.0929	0.0434	−
	S_4_	−	0.5101	0.3862	0.2567	−	0.8589	0.3715	0.1826	0.0959	−
	S_5_	−	0.6057	0.5344	0.4716	0.3024	0.8672	0.5267	0.4410	0.4105	0.1500

**Table 5:** $\rho_{A,B}^{D}$, when D = C, at A, B ∈ S_i_, i = 1, 2,…, 5.

γ	A ∈ S_i_	Cold, B ∈ S_i_				
		S_1_	S_2_	S_3_	S_4_	S_5_
0.1	S_1_	0.1617	−	−	−	−
	S_2_	0.6786	0.1867	0.0442	−	−
	S_3_	0.7356	0.3172	0.1980	0.1424	−
	S_4_	0.8152	0.4204	0.2769	0.2043	−
	S_5_	0.8368	0.5853	0.5140	0.4806	0.2677
0.2	S_1_	0.1364	−	−	−	−
	S_2_	0.6448	0.1584	−	−	−
	S_3_	0.7171	0.3128	0.1709	0.1205	−
	S_4_	0.7970	0.4153	0.2436	0.1763	−
	S_5_	0.8191	0.5674	0.4799	0.4488	0.2395
0.3	S_1_	0.1182	−	−	−	−
	S_2_	0.6291	0.1383	−	−	−
	S_3_	0.7094	0.3059	0.1509	0.1040	−
	S_4_	0.7892	0.4087	0.2197	0.1557	−
	S_5_	0.8108	0.5547	0.4572	0.4277	0.2176
0.4	S_1_	0.1031	−	−	−	−
	S_2_	0.6224	0.1215	−	−	−
	S_3_	0.7068	0.2972	0.1337	0.0896	−
	S_4_	0.7866	0.4011	0.1995	0.1379	−
	S_5_	0.8073	0.5444	0.4402	0.4120	0.1984
0.5	S_1_	0.0893	−	−	−	−
	S_2_	0.6225	0.1063	−	−	−
	S_3_	0.7080	0.2865	0.1177	0.0762	−
	S_4_	0.7879	0.3924	0.1812	0.1213	−
	S_5_	0.8074	0.5353	0.4268	0.3999	0.1801
0.6	S_1_	0.0761	−	−	−	−
	S_2_	0.6292	0.0917	−	−	−
	S_3_	0.7130	0.2734	0.1020	0.0631	−
	S_4_	0.7931	0.3822	0.1634	0.1050	−
	S_5_	0.8110	0.5269	0.4164	0.3911	0.1620
0.7	S_1_	0.0629	−	−	−	−
	S_2_	0.6440	0.0770	−	−	−
	S_3_	0.7230	0.2564	0.0858	0.0497	−
	S_4_	0.8030	0.3696	0.1452	0.0881	−
	S_5_	0.8189	0.5189	0.4092	0.3858	0.1429
0.8	S_1_	0.0488	−	−	−	−
	S_2_	0.6713	0.0610	−	−	−
	S_3_	0.7410	0.2328	0.0679	0.0355	−
	S_4_	0.8201	0.3526	0.1254	0.0695	−
	S_5_	0.8333	0.5110	0.4062	0.3855	0.1216
0.9	S_1_	0.0323	−	−	−	−
	S_2_	0.7253	0.0418	−	−	−
	S_3_	0.7771	0.1937	0.0459	0.0196	−
	S_4_	0.8522	0.3245	0.1008	0.0468	−
	S_5_	0.8619	0.5030	0.4120	0.3957	0.0941

Tables 4 and 5 allow for the conclusion that:

1. Using HDM to improve the set B ∈ S_1_, L (0.1) will increase from 5.3815 to 5.4559, as shown in Table 2. Reducing the set’s failure rate may have the same effects on L (0.1): (i) A ∈ S_1_, by *ρ*^*H*^ = 0.6323, (ii) A ∈ S_2_ when *ρ*^*H*^ = 0.8882, (iii) A ∈ S_3_ when *ρ*^*H*^ = 0.9096, (iv) A ∈ S_4_ when *ρ*^*H*^ = 0.9414, (v) A ∈ S_5_, by the same factor *ρ*^*H*^ = 0.9438, see Table 4.
2. Using ISDM to improve the set B ∈ S_1_, L (0.1) will increase from 5.3815 to 5.5695, as shown in Table 2. Reducing the set’s failure rate may have the same effects on L (0.1): (i) A ∈ S_1_, when *ρ*^*I*^ = 0.2884, (ii) A ∈ S_2_ when *ρ*^*I*^ = 0.7432, (iii) A ∈ S_3_ when *ρ*^*I*^ = 0.7898, (iv) A ∈ S_4_ when *ρ*^*I*^ = 0.8565, (v) A ∈ S_5_, by the same factor *ρ*^*I*^ = 0.8699, see Table 4.
3. Using CDM to improve the set B ∈ S_1_, L (0.1) will increase from 5.3815 to 5.6274, as shown in Table 3. By reducing the set’s failure rate, L(0.1) can be affected in the same method: (i) A ∈ S_1_, when *ρ*^*C*^ = 0.1617, (ii) A ∈ S_2_ when *ρ*^*C*^ = 0.6786, (iii) A ∈ S_3_ when *ρ*^*C*^ = 0.7356, (iv) A ∈ S_4_ when *ρ*^*C*^ = 0.8152, (v) A ∈ S_5_, by the same factor *ρ*^*C*^ = 0.8368, see Table 5.
4. The remaining results shown in Tables 2–5 may be read in the same manner.
5. The symbol ’−’ indicates that the two upgraded systems acquired by the reduction technique and duplication techniques are not equivalent.

## 7 Conclusion

This study presented an evaluation and upgrade of the performance of a bridge network structure modeled with Rayleigh distribution lifetimes. The analysis demonstrated that the performance can be significantly influenced by distinct techniques to increase the systems’ reliability: lowering the failure rate, using imperfect switch redundancy, warm redundancy, and cold redundancy. The use of the bridge structure with identical and independent items with Rayleigh distribution lifetimes provided a more accurate representation of the random failure rates of bridge items, allowing for a more nuanced understanding of the network’s overall reliability. Numerical simulation and the findings indicated that the upgraded design to the most vulnerable items, based on their failure rates, can significantly enhance the overall network performance without necessitating complete overhauls. In conclusion, the proposed methodology for evaluating and upgrading bridge network structures based on Rayleigh lifetime distributions offers valuable insights for engineers and decision-makers. This approach of our techniques ensures that bridge networks can maintain high levels of performance and safety throughout their lifespan, while minimizing costs and disruptions associated with traditional replacement strategies. Future work can expand on these findings by considering additional factors such as failure rate conditions, different distributions, and more complex structure dependencies within network items.
